# Editorial: The impact of environmentally friendly agricultural practices on soil microbiome

**DOI:** 10.3389/fmicb.2024.1505220

**Published:** 2024-10-30

**Authors:** Loredana Canfora, Massimo Pugliese, Ewa Maria Furmanczyk

**Affiliations:** ^1^Council for Agricultural Research and Agricultural Economy Analysis (CREA), Research Centre Agriculture and Environment, Rome, Italy; ^2^Department of Agricultural, Forestry and Food Sciences (DISAFA), University of Turin, Turin, Italy; ^3^AGROINNOVA–Interdepartmental Centre for the Innovation in the Agro-Environmental Sector, University of Turin, Turin, Italy; ^4^Department of Plant Protection, The National Institute of Horticultural Research, Skierniewice, Poland

**Keywords:** plant growth-promoting rhizobacteria (PGPR), biofertilizer, VOCs, biocontrol, fungi

There is a general recognition that intensive agricultural systems are highly energy-dependent and extremely vulnerable to pest outbreaks and climatic variability. The worldwide trend in reducing chemical fertilizers and plant protection products is generating a growing interest in environmentally friendly agricultural practices. These strategies include innovative crop management practices by using different living mulching technologies, which enhance pollinators and other beneficial insects' occurrence and activity, by implementing agricultural practices such as crop rotation and intercropping, and by applying microbial-based products. Using biofertilizers, natural substances and microorganisms as plant biostimulants, resistance inducers and biocontrol agents is becoming more widespread.

In this context, both scientific and regulatory aspects come into play, making it essential to monitor the impact of these sustainable practices on bulk and rhizosphere microorganisms and overall soil and plant health. Various methods can be used to evaluate these impacts, including advanced molecular tools, which offer insights into the ecological dynamics of soil microbiomes.

Current Research Topic efforts are largely focused on assessing the direct effects of these sustainable strategies on plant yield, health status, and economic viability. However, less attention has been given to their indirect effects, such as changes in microbial diversity and activity. These changes are critical indicators of ecological equilibrium, as soil microorganisms play a pivotal role in ecosystem services, including organic matter decomposition, nutrient cycling, and the control of diseases. Despite the crucial importance of these interactions, there remains a significant gap in understanding of how biofertilizers and other microbial introductions affect agroecosystems over time.

The main aim of this Research Topic was to expand knowledge of the role of beneficial microorganisms and their application for crop protection and crop nutrition. Additionally, it aimed to foster innovative approaches that enhance our understanding of sustainable agricultural practices and their impact on soil health. Within this Research Topic, four articles were published, each contributing to our understanding of bacteria and fungi as sustainable solutions for pest management, bioremediation, and biofertilization.

In their review, Almeida et al. emphasizes the role of beneficial microorganisms, such as certain bacteria and fungi, that form mutualistic relationships with plants and can produce volatile organic compounds (VOCs) with biocontrol potential. VOCs, including small molecules like alkenes, alcohols, and terpenes, can inhibit the growth of key pathogens. Genera such as *Bacillus, Pseudomonas, Serratia*, and *Streptomyces* are particularly effective antagonists and VOC-producing microorganisms. VOCs exert their inhibitory effects by disrupting pathogen structures and modulating gene expression. The review underscores the need for further research to clarify these interactions and optimize the use of VOC-producing microorganisms in sustainable agriculture, reducing reliance on agrochemicals.

Hu and Chen contributed to the discussion of the role of heavy metal pollution in agroecosystem. Microbial remediation, mainly through phosphate-solubilizing microorganisms (PSMs), offers a promising and sustainable solution. PSMs not only tolerate high concentrations of HMs but also mitigate their toxicity while enhancing the availability of essential nutrients like phosphorus. Furthermore, they promote plant growth by secreting beneficial metabolites and inhibiting plant pathogens. Continued research is essential to optimize the use of PSMs for HM remediation, ensuring ecological balance and effectiveness. Integrating microbial remediation with other soil management strategies can significantly enhance agricultural sustainability and productivity.

Gen-Jiménez et al. focused on studying the effects of native *Rhizobium* strains on tomato crops. The researchers explored how these strains support plant growth, biofilm formation and root colonization and analyzed changes in the rhizosphere microbial community through metagenomics. The results showed that the native *Rhizobium* strains effectively solubilized dicalcium phosphate, produced essential growth-promoting compounds, and formed biofilms that facilitated root colonization. Inoculating tomato plants with these strains not only improved growth and fruit quality but also altered the composition of the plant microbiome. Metagenomic analysis indicated an increase in the abundance of Proteobacteria and shifts in microbial diversity. Overall, the findings suggest that native *Rhizobium* strains have significant potential as plant probiotics in agriculture. They can contribute to producing safe, high-quality food, enhance soil health, and reduce the environmental impact of chemical fertilizers.

Aslam et al. discussed the potential of using bio-organic phosphate (BOP) fertilizer as a sustainable solution to improve crop yields and reduce reliance on chemical fertilizers. They identified heat-tolerant phosphate-solubilizing bacteria (PSB) from wheat-growing regions in southern Punjab, India. Five of these bacteria showed significant phosphate-solubilizing activity, especially at high temperatures, leading to a decrease in pH. These effective PSB were combined to form groups that demonstrated improved phosphorus solubilization. In a microcosm study the BOP formulation increased total phosphorus levels by 14% compared to uninoculated controls, with plant-based BOP showing higher viable counts than filter mud-based BOP. This study emphasizes the innovative use of bio-organic phosphate fertilizer combined with heat-tolerant PSB, providing an eco-friendly alternative for achieving better wheat yields with lower fertilizer inputs.

In conclusion, the research presented in this Research Topic illustrates the potential of environmentally friendly agricultural practices to influence and improve the soil microbiome while offering a sustainable alternative to intensive farming methods. Despite the advances highlighted by these studies, it remains challenging to provide a comprehensive understanding of the overall impact of such practices on the soil microbiome. Current efforts are largely driven by exploring the innovative use of microbial-based solutions and their direct impact on crop yield and health. However, it is imperative to also focus on the broader ecological impacts—particularly the shifts in microbial diversity and functionality that these practices induce.

To achieve this understanding, future research should prioritize long-term field studies that explore the interactions of beneficial microorganisms within the broader agricultural ecosystem under real-world conditions. The broader adoption of these practices is critical for transitioning to a sustainable agricultural paradigm. By focusing on the role of beneficial microorganisms, we can enhance soil health, reduce dependence on chemical inputs, and promote ecological balance. Projects like EXCALIBUR and similar EU initiatives are vital for building the necessary knowledge base to understand the environmental impact and legacy of these practices, ultimately helping to create a future of resilient and sustainable agriculture.

